# Increased interhemispheric functional connectivity after right anodal tDCS in chronic non-fluent aphasia: preliminary findings

**DOI:** 10.3389/fnins.2024.1346095

**Published:** 2024-02-09

**Authors:** Federica Alemanno, Davide Fedeli, Alessia Monti, Elise Houdayer, Pasquale Anthony Della Rosa, Federica Zangrillo, Daniele Emedoli, Elisabetta Pelagallo, Massimo Corbo, Sandro Iannaccone, Jubin Abutalebi

**Affiliations:** ^1^Neuropsychology Service, Department of Rehabilitation and Functional Recovery, San Raffaele Scientific Institute, Vita-Salute San Raffaele University, Milan, Italy; ^2^Centre for Neurolinguistics and Psycholinguistics, Scientific Institute San Raffaele, Vita-Salute San Raffaele University, Milan, Italy; ^3^Department of Neuroradiology, Fondazione IRCCS Istituto Neurologico Carlo Besta, Milan, Italy; ^4^Department of Neurorehabilitation Sciences, Casa di Cura Igea, Milan, Italy; ^5^Department of Neuroradiology, IRCCS San Raffaele Scientific Institute, Milan, Italy

**Keywords:** aphasia, transcranial direct current stimulation, resting state fMRI, language, stroke

## Abstract

**Introduction:**

Anodal transcranial Direct Current Stimulation (tDCS) is a non-invasive, low-cost and environment-friendly brain neuromodulation technique that increases cortical excitability. In post-stroke aphasia, the role of the right hemisphere in language recovery remains debated. In this preliminary study, we aimed to investigate the efficacy of excitatory tDCS on the right hemisphere in chronic aphasic patients.

**Methods:**

We applied anodal tDCS to the right homologous region of Broca’s area in four chronic aphasic patients while performing a one-month naming rehabilitation treatment. Longitudinal data on language assessment and naming performance were collected. Resting-state fMRI images were acquired before and after treatment to measure changes in functional connectivity.

**Results:**

Results showed enhanced positive functional connectivity of the right Broca homologous with the left middle frontal and middle temporal gyri. Every patient showed improvements in language functions, but no major changes in naming performance.

**Conclusion:**

These preliminary findings suggest that tDCS applied over the unaffected hemisphere may result in longitudinal inter-hemispheric functional neuroplastic changes that could specifically improve language recovery and could potentially be included in therapeutic neurorehabilitative plans.

## Introduction

Post-stroke language recovery in aphasic patients is a complex and dynamic process influenced by many factors, including initial aphasia severity, brain lesion site and size, etiology, elapsed time from the stroke onset and the specific type of rehabilitation (Gerstenecker and Lazar, 2019). Transcranial Direct Current Stimulation (tDCS) has been used in clinical treatments and post-stroke aphasia rehabilitation protocols to prompt neuroplastic changes in the lesioned brain activity. A growing number of studies successfully applied excitatory (anodal) or inhibitory (cathodal) tDCS in combination with speech and language therapy and showed enhanced language recovery after treatment (Harvey and Hamilton, 2022). Most treatments applied anodal tDCS (A-tDCS, excitatory) on the left hemisphere (LH) in uni-cephalic montages. Other studies have focused on inhibiting the allegedly maladaptive activation of the RH using cathodal tDCS (C-tDCS) (Vines et al., 2009, 2011; Turkeltaub, 2015). Only few experimental designs have applied excitatory A-tDCS on the RH to promote functional reorganization. Vines and colleagues (Vines et al., 2011) reported improvement in verbal fluency after A-tDCS neuromodulation of the RH homologous of the lesioned left inferior frontal gyrus (IFG) in conjunction with Melodic Intonation Therapy (MIT), which facilitates language recovery. Flöel et al. (2011) administered A-tDCS (excitatory), C-tDCS (inhibitory) and sham tDCS (placebo stimulation) over the RH temporoparietal cortex in aphasic patients, while performing anomia training. The behavioral outcome resulted in enhanced performance in object naming in all the conditions.

In this preliminary study, we used resting state functional connectivity to investigate the efficacy of excitatory tDCS on the right homologous region of Broca’s area and the underlying mechanisms of neural plasticity in a small group of chronic aphasic patients.

## Materials and methods

### Participants

Four patients were included in the study (all males; mean age = 69 years; SD = 2.71; time elapsed from the stroke onset was at least 6 months—mean = 11.25; SD = 8.54) (see Table 1 for demographic and clinical characteristics of the study participants). All patients were diagnosed with chronic non-fluent aphasia with persistent and severe naming deficits through the Italian version of the Aachener Aphasie Test (AAT) (Luzzatti et al., 1994). The AAT examines the verbal performances in comprehension and expression, in the oral and written modality. The subtests are composed of 3–5 item sets, of 10 items each, for the examination of different linguistic units (phonemes, simple lexemes, complex lexemes produced according to morphological combination rules, and sentences). Individual item content is graded according to specified criteria of linguistic complexity, in order to differentiate aphasic disorders for a wide range of severity (Luzzatti et al., 2023).

**Table 1:** Demographic and clinical characteristics of the study participants.

ID	Age	Gender	Education	Time from stroke (months)	Lesion side (hemisphere)	Lesion volume (ml)-MNI	Lesion- Driven ICs (pre)	Lesion- Driven ICs (post)	AICHA regions with 95% damage
S1	71	M	8	24	Left	264.26	16	18	27
S2	65	M	8	8	Left	37.84	3	3	1
S3	70	M	13	7	Left	189.31	3	4	4
S4	70	M	13	6	Left	193.17	7	7	13

Number of lesion-driven Independent Components (ICs) and massively damaged AICHA regions are also reported for each subject.

Individual raw scores and corresponding severity ranges (severe, moderate, mild, light) for repetition, written language, naming and comprehension were reported for each patient. Spontaneous speech was evaluated only in two patients (1 and 4), because of lack of spontaneous production in the other two. The study was approved by our local Ethic Committee and participants gave their informed consent, according to the Declaration of Helsinki.

### Cases description

#### Patient 1

Seventy-one y.o. right-handed man who suffered from an ischemic stroke resulting in a massive left hemispheric lesion extended over a large network of cortical and subcortical regions. The Automated Anatomical Labeling (AAL) atlas (Tzourio-Mazoyer et al., 2002) was used to define left hemispheric regions damaged at least at 50% of their volume: Inferior Frontal Gyrus (Pars Opercularis, Pars Triangularis); Rolandic Operculum; Insula; Inferior Parietal Lobule; Supramarginal Gyrus; Angular Gyrus; Putamen; Heschl’s Gyrus; Superior Temporal Gyrus; Middle Temporal Gyrus. Patient 1’s aphasia presented with mixed features. The patient was non-fluent and right hemiplegic. AAT scores showed a profile of non-fluent aphasia, with impairment in spontaneous speech, anomia, phonological errors and word fragments. Repetition, naming, written and oral comprehension were also moderately compromised. Reading aloud words and phrases was not evaluated.

#### Patient 2

Sixty-five y.o. right handed man who suffered from ischemic stroke after left internal carotid artery dissection (MR and CT Angiography) following a traumatic accident resulting in left posterior temporal and subcortical (mostly lenticular) lesions. Even if these lesions were much smaller compared with those of the other patients, the clinical assessment revealed a severe form of non-fluent aphasia, right hemiplegia and hemianopsia and was diagnosed by a neurologist with Broca’s aphasia. AAL regions damaged at least at 50% were the left Putamen and the left Globus Pallidus, with less damage over temporal and insular cortices. The patient had a very severe buccofacial apraxia. Repetition, written language and naming were severely impaired, while comprehension was compromised to a lesser extent.

#### Patient 3

Seventy y.o. right-handed man who suffered from a left middle cerebral artery (MCA) ischemic stroke, resulting in non-fluent aphasia, dysphagia, right spastic hemiplegia and buccofacial apraxia. The left frontal-temporal-insular lesion revealed by MR scan involved both cortical and subcortical areas. AAL regions damaged at least at 50% were: left Inferior Frontal Gyrus (Pars Opercularis); left Supramarginal gyrus; left Putamen; left Inferior Temporal gyrus. Patient 3’s aphasia presented with mixed features. The patient had severe deficits in naming (with prevalence of anomia, phonological errors and presence of perseveration) but had a spared prosody. Repetition, comprehension (both auditory and written) and written language were impaired. More specifically, the performance in word copying and in letter matching was severely impaired.

#### Patient 4

Seventy y.o. right-handed man who suffered from a left MCA occlusion, resulting in right hemiparesis and non-fluent aphasia. MRI and two CT scans showed a left frontal-temporal- insular lesion involving both cortical and subcortical (lenticular) areas. Left hemispheric AAL regions damaged at least at 50% were: Precentral Gyrus; Inferior Frontal Gyrus (Pars Opercularis; Pars Triangularis); Rolandic Operculum; Insula; Supramarginal Gyrus; Putamen; Heschl’s Gyrus; Superior Temporal Gyrus; Middle Temporal Gyrus. Patient 4’s aphasia presented with mixed features. The patient had severely compromised performance in naming (anomia), repetition and written comprehension, although auditory comprehension was only moderately impaired. Written language was completely damaged.

### Study design and rehabilitation treatment

Treatment consisted in daily naming training sessions, 5 days a week, for 4 consecutive weeks. Each session lasted 45 min. During the first 20 min, anodal tDCS was administered simultaneously to picture naming training. A-tDCS was delivered by a constant-current stimulator (HDC-kit Newronika s.r.l. Milan, Italy) with a 1.5 mA intensity, through a pair of saline-soaked sponge electrodes (size = 5 cm × 7 cm; current density = 0.04 mA/cm^2^), anode over the right homologous region of Broca’s area (corresponding to the right inferior prefrontal cortex, the electrode was centered in the midline between F6-F8 of the international 10/20 system for EEG electrodes placement; Scrivener and Reader, 2022) and reference electrode (cathode) over the left deltoid.

Language deficits were assessed with the AAT and the Snodgrass and Vanderwart picture naming test which included a set of 260 pictures of concrete objects (e.g., animals, tools, vegetables) (Snodgrass and Vanderwart, 1980). Treatment consisted in naming pictures showed by a computer. At the end of the 4-week treatment, patients were re-tested with the AAT and picture naming of all the figures, including the unnamed items.

Statistical analyses of the pre and post scores were performed using Wilcoxon non-parametric test. Data were considered significant when *p* ≥ 0.05. The commercially available software IBM SPSS Statistics v.23 (IBM Corp. ©) was used for statistical testing.

### MRI acquisition

MRI scanning was performed before and after the four-week treatment with a 3 T Philips Ingenia CX MR scanner (Philips Medical Systems, Best, Netherlands) using a 32 channels SENSE head coil. In each run, a high-resolution MPRAGE (Magnetization Prepared Rapid Gradient Echo) T1-weighted anatomical image was acquired [Echo Time (TE) = 4 ms; Repetition Time (TR) = 8 ms; Flip Angle (FA) = 8°; Field of View (FOV) = 256 × 256 mm^2^; matrix size = 230 × 230; 150 axial slices; slice thickness = 2 mm; interslice gap = 1 mm; voxel size = 1 × 1 × 1 isotropic; PE direction = R/L; whole brain coverage]. Additionally, 7 min Resting-State functional scans were acquired with a fast speed echo Echo Planar Imaging (EPI) sequence [TE = 20 ms; TR = 2,500 ms; FA = 85°; number of volumes = 150; FOV = 240 × 240 mm^2^; matrix size = 84 × 84; 54 axial slices per volume; slice thickness = 3; interslice gap = 3; voxel size = 2.5 × 2.5 × 3; Phase Encoding direction (PE) = A/P; SENSE factor = 2; whole brain coverage]. Ten dummy scans preceded each run to optimize EPI image signal.

### Preprocessing

#### Preprocessing of structural scans

Each subject’s structural T1 images were first oriented to match the AC-PC line and then aligned to each other with SPM12 v6685. Brain lesions were automatically identified with the Lesion Identification with Neighborhood Data Analysis (LINDA) toolkit, based on the Advanced Normalization Tools ANTs running on R (R Core Team, 2013). Binarized lesion masks in both native and in MNI space were computed and visually inspected to verify the correct lesion identification.

Enantiomorphic normalization was performed using the Clinical Toolbox. First, the T1 image was midline-aligned and mirrored over the lesioned hemisphere. This new reflected image was then co-registered to the native T1. The lesioned tissue was replaced by the corresponding tissue from the mirrored scan to create a chimeric image. This chimeric image was then normalized through the unified segmentation-normalization procedure. The resulting deformation fields were applied both to the native T1 scan and to the lesion mask.

The inverse spatial transform was used to create subject-specific native space regions of interest (ROIs) maps from the atlas of intrinsic connectivity of homotopic areas (AICHA) developed by Joliot et al. (2015), which segments the grey matter into 384 ROIs. Brain damage was then estimated as the number of regions interested by the lesion mask (95% volume overlap) (see Table 1). AAL atlas was also used to define anatomical regions damaged at least at 50% to more clearly express the structural damage.

#### Preprocessing of fMRI scans

First, functional images were slice time corrected, and then images were realigned and unwarped for motion and geometric distortions correction (Yourganov et al., 2017). Data from each session were then coregistered to the relative structural image. Finally, the images were spatially smoothed with a Gaussian Kernel with Full-Width at Half-Maximum (FWHM) = 6 mm. Preprocessing was done with SPM 12. Individual brain masks were estimated for each subject. Processed fMRI timecourses were entered into the CONN toolbox v17f (Whitfield-Gabrieli and Nieto-Castanon, 2012) and detrended via CompCor strategy using the mean signal from the white matter, obtained from the chimeric T1-weighted image; the six motion parameters from the realignment step; ramping effects at the beginning of each scanning session; linear, quadratic and cubic trends. We then applied a 0.01–0.1 Hz bandpass-filter to remove low-frequency drift and high-frequency noise. Global mean signal and chimeric cerebrospinal fluid (CSF) signal were not regressed out. Following the “Pipeline C” method (Yourganov et al., 2017), we used the nii_filter_lesion_ICs.m MATLAB script that identifies lesion-derived independent components and then regresses them out of the fMRI data. We performed probabilistic Independent Component Analysis (pICA) (Beckmann and Smith, 2004) on the previously denoised data, using Multivariate Exploratory Linear Optimized Decomposition into Independent Components (MELODIC) version 3.15, part of FSL. Previously estimated individual brain masks were used as a reference. The Z-scored IC spatial maps were thresholded at *p* < 0.05 and overlapped to the lesion masks of the patients. Significantly overlapping ICs (*p* < 0.05) were then regressed out using fsl_regfilt FSL script (number of ICs are reported in Table 1). ICA-filtered data were then re-entered into the CONN toolbox as raw data, skipping the initial preprocessing and denoising steps. BOLD time series were computed for each ROI of the AICHA atlas, masked by individual grey matter maps. First level ROI-to-ROI correlation analyses were used to determine the linear association of the BOLD time series between each pair of sources and a Fisher Z transformation was applied to the estimated R correlation coefficients. 384×384 ROI-to-ROI connectivity matrices were then computed for both the pre-rehabilitation and the post-rehabilitation conditions. ROI-to-ROI maps were entered into the second-level analysis to measure, group wise, the effect of rehabilitation on brain connectivity.

#### Resting state analysis

A between-session *T*-Test was performed to investigate directional treatment-induced differences in functional connectivity generalized across all the subjects. In order to examine post-treatment > pre-treatment effects and specifically investigate the supporting role of the right hemisphere following inferior PFC stimulation, we constrained our analyses to inter-hemispheric effects (target ROIs only in the LH) selecting three ROIs in the right IFG as seeds (S_Inf_Frontal-1-R; S_Inf_Frontal-2-R; and G_Frontal_Inf_Tri-1-R), with MNI centroid coordinates corresponding to right BA 44 and 45. This analysis allows to measure connectivity changes of the stimulated regions with the lesioned areas of the brain, thus helping to directly explore the strength and direction of the effect of excitatory stimulation of the contralateral hemisphere on the lesioned LH. Results were thresholded with a *p* < 0.05 threshold with a False Discovery Rate (FDR) analysis level correction for multiple comparisons. Finally, we explored between-ROI connectivity by means of Network Based Statistics (NBS) (Zalesky et al., 2010) with 10,000 iterations and applying a statistical threshold of *p* < 0.05 with Family Wise Error (FWE) correction for multiple comparisons.

## Results

### Neuropsychological tests

Aachener Aphasie Test and Snodgrass and Vanderwart picture naming scores are reported in Table 2. Every patient improved in the repetition and comprehension AAT subtests, although such result did not reach significance. Only a tendency for improvement was observed, for both repetition and comprehension results (*p* = 0.068 and *p* = 0.066, respectively).

**Table 2:** Neuropsychological tests: pre and post treatment AAT and Snodgrass and Vanderwart picture naming scores.

	S1				S2				S3				S4			
	Pre-treatment		Post-treatment		Pre-treatment		Post-treatment		Pre-treatment		Post-treatment		Pre-treatment		Post-treatment	
AAT	Raw score	Severity	Raw score	Severity	Raw score	Severity	Raw score	Severity	Raw score	Severity	Raw score	Severity	Raw score	Severity	Raw score	Severity
Spontaneous language	1/5		3/5										0/5		0/5	
Communicative behavior	2/5		3/5										1/5		1/5	
Articulation and prosody	5/5		5/5										2/5		2/5	
Formulaic language	2/5		2/5										0/5		0/5	
Lexico-semantics	1/5		2/5										0/5		0/5	
Phonology	4/5		4/5										0/5		0/5	
Syntax	0/5		0/5										0/5		0/5	
Repetition	104/150	Moderate	113/150	Moderate	35/150	Severe	40/150	Severe	53/150	Severe	78/150	Moderate	49/150	Severe	92/150	Moderate
Reading aloud words/phrases	NE	NE	13/30	Moderate	2/30	Severe	1/30	Severe	0/30	Severe	0/30	Severe	0/30	Severe	0/30	Severe
Composing words/phrases from blocks	8/30	Moderate	14/30	Moderate	5/30	Moderate/severe	5/30	Moderate/severe	NE	NE	NE	NE	NE	NE	NE	NE
Writing words/phrases to dictation	6/30	Moderate	9/30	Moderate	3/30	Moderate/severe	6/30	Moderate/severe	0/30	Severe	0/30	Severe	NE	NE	NE	NE
Naming	66/120	Moderate	78/120	Moderate	3/120	Severe	2/120	Severe	0/120	Severe	3/120	Severe	3/120	Severe	9/120	Severe
Auditory comprehension	44/60	Moderate	52/60	Mild	45/60	Moderate/mild	53/60	Mild	23/60	Severe	36/60	Severe	43/60	Moderate	48/60	Mild
Reading comprehension	32/60	Moderate	31/60	Moderate	44/60	Moderate/mild	44/60	Moderate/mild	17/60	Severe	30/60	Moderate	11/60	Severe	12/60	Severe
Naming																
Snodgrass and Vanderwart (1980)	146/260		133/260		0/260		0/260		0/260		5/260		10/260		27/260	

NE, not performed.

Three patients (patients 1, 3 and 4) showed a better or similar performance in naming new untrained stimuli after the one-month training, although such result did not reach significance (*p* > 0.05).

### Resting state functional connectivity

**Figure 1:**
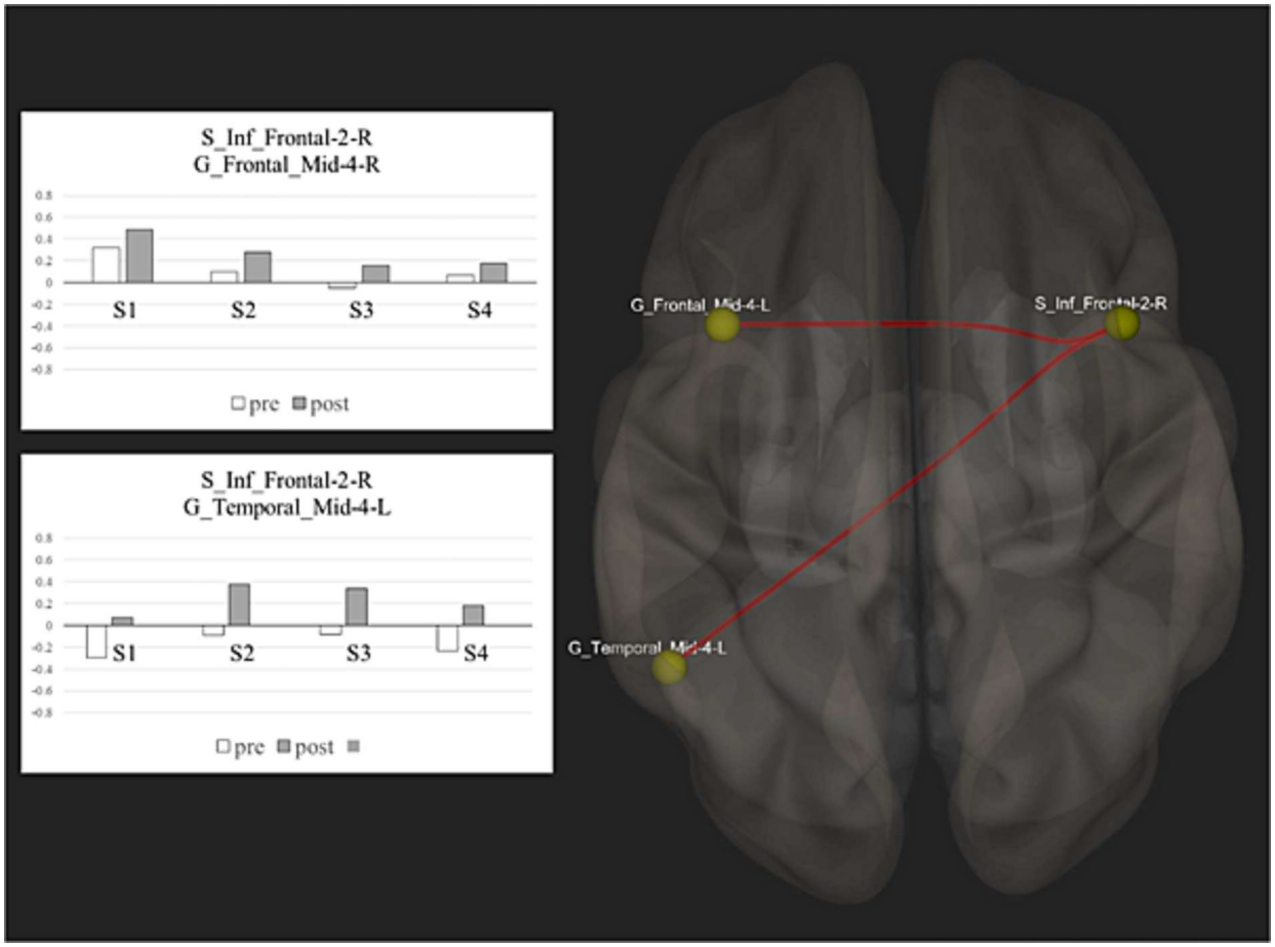
Resting state functional connectivity: this figure shows the spatial distribution of the network nodes and the correlation scores before and after treatment between the significant ROIs activity.

Our results showed a significant change in functional connectivity, with enhanced positive connectivity between the right Inferior Frontal Sulcus (S_Inf_Frontal-2-R) and the left Middle Frontal Gyrus (G_Frontal_Mid-4-L) [T(3) = 18.80; *p* = 0.0002] and between the right Inferior Frontal Sulcus (S_Inf_Frontal-2-R) and the posterior part of the left Middle Temporal Gyrus (G_Temporal_Mid-4-L) [T(3) = 17.53; *p* = 0.0002] (see Figure 1). The between-ROI network showed a significant increase of functional connectivity after treatment (size = 4; intensity = 72.66; *p* < 0.001).

## Discussion

After excitatory A-tDCS over the right homologous region of Broca’s area, patients showed a tendency for improvement in comprehension and repetition abilities. Snodgrass and Vanderwart naming generalization performance was not consistent among all the patients, but three out of four patients (patients 1, 3, and 4) were able to name new untrained stimuli, possibly hinting an effect of generalization. Interestingly, the three patients who could perform effective naming generalization were those with more extended brain lesions, including severe damage of the left IFG (>50% of the AAL Pars Triangularis and Opercularis regions). Many studies suggested that the hyperexcitability observed in the RH after LH stroke would compensate for part of the damaged functions (Basso et al., 1989; Gainotti, 1993, 2015; Anglade et al., 2014). Excitatory A-tDCS stimulation over the right homologous region of Broca’s area conjointly with naming training might have had a beneficial effect on the behavioral performance of those patients with wide lesions including the left IFG. These claims are coherent with the connectivity results from resting state fMRI analysis. We found significantly increased interhemispheric connectivity between the seed region (right IFG) stimulated by A-tDCS, and two target areas located, respectively, in the left Middle Frontal Gyrus (MFG) and left Middle Temporal Gyrus (MTG). In post-stroke patients, behavioral impairment is frequently associated with disrupted interhemispheric connectivity that can affect distant regions and, more specifically, with reduced connectivity between homotopic areas (Grefkes and Fink, 2014; Baldassarre et al., 2016; Siegel et al., 2016; Klingbeil et al., 2019). Marangolo et al. (2016) investigated the effects of bi-cephalic tDCS (anode over left IFG, cathode over right IFG) and speech therapy on functional connectivity in aphasic patients, showing increased LH intrahemispheric connectivity. Our study inspected the impact of excitatory A-tDCS applied over the right homologous region of Broca’s area in aphasia rehabilitation. We showed significant changes in the functional connectivity proprieties of the selected target stimulated region, which is contrasting with the model of Transcallosal Inhibition. If the model held true, an excitatory stimulation would have facilitated cortical activation of the RH and therefore increased the detrimental inhibition of the LH. On the contrary, enhanced positive interhemispheric connectivity was found. Moreover, according to the Transcallosal Inhibition hypothesis (Netz et al., 1995), the maladaptive activation of the RH would have led to a decreased behavioral performance, which was not observed in this study. Indeed, repetition and comprehension equivalent scores increased after treatment in all patients, without reaching significance at the statistical analyses. Only a tendency for improvement was observed, for both repetition and comprehension results. The naming generalization performance also improved for 3 out of 4 patients, although it did not reach statistical significance. This latter result differs from previously reported in the literature (Flöel et al., 2011). However, it is important to note that in the study of Flöel et al., the speech training lasted 2 h per day. Significant improvements in naming were observed, also in the sham group, suggesting that the training itself might have been efficient enough to induce naming improvements, as it has been largely reported that the efficacy of language therapy depends on treatment intensity (Hartwigsen and Saur, 2019). It should also be pointed out that the two target areas (left MFG and left MTG) which resulted significantly more connected to the right stimulated region are spatially neighboring the damaged Broca and Wernicke areas in the left hemisphere. According to the model presented by Anglade et al. (2014), in efficient recovery, the functional language activity moves to the regions close to the lesion. The increased interhemispheric connectivity with these target regions could potentially clarify the improvement in repetition and comprehension scores. Patients were in a chronic stage (>6 months). In this phase, language functions’ recovery is mostly linked to the therapeutic intervention, while spontaneous recovery is minimal (Yamaji et al., 2021). Thus, we might hypothesize that neuroplasticity changes that we measured would be related to the stimulation/rehabilitation procedure and not to spontaneous recovery. Our results fit with the scheme of the mechanisms of disruption and reorganization in the language network proposed by Hartwigsen and Saur (Hartwigsen and Saur, 2019). They suggested that at chronic stages, neural plasticity reorganization would result in increased interhemispheric connectivity between the bilateral frontal areas and within the left frontal and left temporal regions. Furthermore, according to the authors, this network could also be effectively modulated by non-invasive brain stimulation. It is also probable that the picture naming training itself might have induced such neuroplastic changes as language therapy can significantly improve language functions in the chronic phase after stroke (Breitenstein et al., 2017). Previous studies reported increased activity in the right frontal cortex (Blasi et al., 2002) or in the right superior temporal gyrus and the left precuneus (Musso et al., 1999) after language training in chronic stroke patients, strongly supporting the compensatory role of the right hemisphere. Our results are in accordance with the hypothesis that both activation of homologous contralesional cortex and restoration of activity of left hemisphere regions (perilesionally and at distance) would constitute viable mechanisms for language recovery Blasi et al. (2002).

This preliminary study shows increased inter-hemispheric connectivity following excitatory A-tDCS stimulation over the right homologous region of Broca’s area in chronic aphasic patients, suggesting a possibly beneficial effect of excitatory stimulation of the preserved hemisphere in chronic patients with extensive LH lesions. Therefore, future studies, including a wider sample of patients, a more intense daily training, a control group and groups with different tDCS montages should be carried out to provide stronger evidence to these promising results.

## Data availability statement

The raw data supporting the conclusions of this article will be made available by the authors, without undue reservation.

## Ethics statement

The studies involving humans were approved by the IRCCS San Raffaele Ethics Committee. The studies were conducted in accordance with the local legislation and institutional requirements. The participants provided their written informed consent to participate in this study.

## Author contributions

FA: Investigation, Methodology, Writing – original draft. DF: Formal analysis, Investigation, Writing – original draft. AM: Formal analysis, Investigation, Writing – review & editing. EH: Methodology, Writing – review & editing. PD: writing – review & editing. FZ: writing – review & editing. DE: writing – review & editing. EP: writing – review & editing. MC: Resources, Supervision, Writing – review & editing. SI: Resources, Supervision, Writing – review & editing. JA: Conceptualization, Supervision, Writing – review & editing.
